# Mapping the Distribution of Anthrax in Mainland China, 2005–2013

**DOI:** 10.1371/journal.pntd.0004637

**Published:** 2016-04-20

**Authors:** Wan-Jun Chen, Sheng-Jie Lai, Yang Yang, Kun Liu, Xin-Lou Li, Hong-Wu Yao, Yu Li, Hang Zhou, Li-Ping Wang, Di Mu, Wen-Wu Yin, Li-Qun Fang, Hong-Jie Yu, Wu-Chun Cao

**Affiliations:** 1 The State Key Laboratory of Pathogen and Biosecurity, Beijing Institute of Microbiology and Epidemiology, Beijing, People’s Republic of China; 2 Division of Infectious Disease, Key Laboratory of Surveillance and Early-warning on Infectious Disease, Chinese Centre for Disease Control and Prevention, Beijing, People’s Republic of China; 3 Department of Geography and Environment, University of Southampton, Southampton, United Kingdom; 4 Flowminder Foundation, Stockholm, Sweden; 5 Department of Biostatistics, College of Public Health and Health Professions, and Emerging Pathogens Institute, University of Florida, Gainesville, Florida, United States of America; University of California San Diego School of Medicine, UNITED STATES

## Abstract

**Background:**

Anthrax, a global re-emerging zoonotic disease in recent years is enzootic in mainland China. Despite its significance to the public health, spatiotemporal distributions of the disease in human and livestock and its potential driving factors remain poorly understood.

**Methodology/Principal Findings:**

Using the national surveillance data of human and livestock anthrax from 2005 to 2013, we conducted a retrospective epidemiological study and risk assessment of anthrax in mainland China. The potential determinants for the temporal and spatial distributions of human anthrax were also explored. We found that the majority of human anthrax cases were located in six provinces in western and northeastern China, and five clustering areas with higher incidences were identified. The disease mostly peaked in July or August, and males aged 30–49 years had higher incidence than other subgroups. Monthly incidence of human anthrax was positively correlated with monthly average temperature, relative humidity and monthly accumulative rainfall with lags of 0–2 months. A boosted regression trees (BRT) model at the county level reveals that densities of cattle, sheep and human, coverage of meadow, coverage of typical grassland, elevation, coverage of topsoil with pH > 6.1, concentration of organic carbon in topsoil, and the meteorological factors have contributed substantially to the spatial distribution of the disease. The model-predicted probability of occurrence of human cases in mainland China was mapped at the county level.

**Conclusions/Significance:**

Anthrax in China was characterized by significant seasonality and spatial clustering. The spatial distribution of human anthrax was largely driven by livestock husbandry, human density, land cover, elevation, topsoil features and climate. Enhanced surveillance and intervention for livestock and human anthrax in the high-risk regions, particularly on the Qinghai-Tibetan Plateau, is the key to the prevention of human infections.

## Author Summary

Anthrax is a worldwide zoonosis affecting mostly grazing herbivores, with occasional spillover to humans who have contact with infected animals or contaminated animal products. We characterized the distributional patterns of both human and livestock anthrax in China from 2005 to 2013, and identified agro-ecological, environmental and meteorological factors contributing to the temporal and spatial distributions of the disease. We found that the spatial distribution of human anthrax in China was mainly driven by densities of cattle, sheep and humans, coverage of meadow, coverage of typical grassland, elevation, pH level of topsoil, concentration of organic carbon in topsoil, and meteorological factors. We also identified the regions with higher probabilities for the occurrence of human cases. Our findings provided a clear qualitative and quantitative understanding of the epidemiological characteristics and risk recognition of anthrax in China, and can be helpful for prioritizing surveillance and control programs in the future.

## Introduction

Anthrax is one of the ancient zoonoses caused by *Bacillus anthracis* [1]. It is primarily a disease in herbivores and sometimes sparks outbreaks in human with potentially serious consequences [2]. It is enzootic in most countries in Africa and Asia as well as some countries in Europe and America [3]. The disease occurs worldwide with an estimate of 20,000 to 100,000 new human cases each year [4]. According to the World Health Organization (WHO), developing countries in Africa and those in central and southern Asia have the highest human incidences of naturally occurred anthrax [5]. Because of its wide distribution and its potential use for bioterrorism, anthrax is considered as a global public health threat [6]. Concerns have been heightened by the persistent existence of human anthrax cases and outbreaks across continents in recent years, e.g., Zambia, Zimbabwe and Ethiopia in Africa [7–9], India, Bhutan, Bangladesh and Georgia in Asia [10–14], and Turkey, Greece and Serbia in Europe [15–17]. In addition, the emergence of “injectional anthrax” among heroin users in Europe highlights the possibility of new routes for the spread of human anthrax [18, 19].

*Bacillus anthracis*, the causative agent of anthrax, is a sporulating Gram-positive bacterium that manifests a particular bimodal lifestyle: the vegetative phase and the spore phase [2]. Bacteria in the vegetative phase are shed by infected animals and may die rapidly in most environmental conditions. Once sporulated from the vegetative cells, the bacteria can survive in soil for decades [20]. It has been speculated that levels of pH and calcium cation in the soil play an important role in the process of germination or in maintaining spore’s viability. Besides, the organic matter in the soil affects spore adhesion [5, 21, 22]. As a result, topsoil conditions may geographically regulate the distribution of anthrax infections. Some other environmental factors including climatic conditions could also be associated with anthrax infection in herbivores and humans [21, 23]. Herbivores, the primary hosts of this pathogen, are usually infected anthrax by ingestion of spores while grazing or browsing [5]. Human infection was usually a result of contacting ill animals during agricultural activities or processing contaminated animal products [24, 25]. Limited person-to-person transmission has been reported. Human anthrax cases are classified into three forms according to the transmission route: the cutaneous form accounts for about 95% of all human cases worldwide, the gastrointestinal form, and the inhalational form.

More than 112 thousands human cases have been reported in China from 1956 to 1997 with three large-scale outbreaks in the years of 1957, 1963 and 1977, respectively [26]. In the recent decades, human and livestock anthrax outbreaks have been reported in many provinces across the nation, such as Liaoning, Inner Mongolia Autonomous Region, Jiangsu, Guizhou, and Xinjiang Autonomous Region [27–31]. Previous studies mainly focused on local case-report, outbreak investigations, or the spatial and temporal distribution of cutaneous anthrax of human cases in China [27–33]. Using the national surveillance data of human and livestock anthrax from 2005 to 2013, we conducted a comprehensive and in-depth retrospective epidemiological study on the spatiotemporal dynamics and risk determinants of anthrax in mainland China.

## Materials and Methods

### Ethical statement

The National Health and Family Planning Commission of China considers the collection of data from human and livestock anthrax cases as part of its routine surveillance, and such data collection is therefore exempt from approval by the institutional review board.

### Data collection and management

In China, inhalational anthrax is managed as a class A infectious disease, while other forms of human anthrax are listed as one of the class B infectious diseases. Cases diagnosed at medical institutions were reported to the Chinese Centre for Disease Control and Prevention (CCDC) through the web-based national Notifiable Infectious Diseases Reporting Information System (NIDRIS) (http://www.cdpc.chinacdc.cn/UVSSERVER2.0/login?fromSmp=true&fromCDC3=true&service=http%3A%2F%2Fwww.cdpc.chinacdc.cn%2Fportal%2FcasAuthUser%3Fvsite%3Dguojia). All clinically-diagnosed and laboratory-confirmed cases during 2005–2013 were included in this study. Routine surveillance of livestock anthrax is conducted by the Ministry of Agriculture of the People's Republic of China. The surveillance data are published monthly on the Official Veterinary Bulletin (http://www.moa.gov.cn/zwllm/tzgg/gb/sygb/), from which we extracted the monthly numbers of livestock cases and outbreaks as well as affected species in each province during the study period. The case definitions for human and livestock anthrax are stated in the S1 Text.

Data concerning agro-ecological, environmental and meteorological factors were collected for exploring potential determinants for the temporal and spatial distributions of human anthrax in mainland China (S1 Table). Raster-typed data with a 5 km^2^ resolution regarding the density of cattle, sheep and goats were obtained from the Food and Agriculture Organization of the United Nations (http://www.fao.org/AG/againfo/resources/en/glw/GLW_dens.html). The human population size and the annual number of livestock including cattle, sheep, goats, pigs and horses were obtained from the National Bureau of Statistics of China (http://www.stats.gov.cn/). The land cover data with a 1 km^2^ resolution in 2005 was collected from the Data Sharing Infrastructure of Earth System Science (http://www.geodata.cn). The elevation data were obtained from Global digital elevation data products (http://www.geodata.cn/data/datadetails.html?dataguid=201519481253546&docid=1301). The soil-related variables including pH level, concentration of organic carbon, and concentration of calcium in topsoil with a 1 km^2^ resolution were derived from the Harmonized World Soil Database (http://webarchive.iiasa.ac.at/Research/LUC/External-World-soil-database/HTML/). The climatic variables including monthly average temperature and relative humidity, as well as monthly accumulative rainfall and sunshine hours during the study period were obtained from the China Meteorological Data Sharing Service System (http://data.cma.cn/).

Using the spatial analytic methods in the ArcGIS 9.2 software (ESRI Inc., Redlands, CA, USA), we extracted the following 15 variables for each county: densities of cattle, sheep, goats and human, percentage coverages of meadow, typical grassland and alpine steppe, average elevation, percentage coverage of topsoil with pH > 6.1, average concentration levels of organic carbon and calcium in topsoil [21], monthly average temperature, relative humidity, and yearly accumulative rainfall, sunshine hours during the study period.

### Analysis of distributional patterns of human and livestock anthrax

Monthly numbers of human cases were plotted to display the seasonal dynamic of the disease. Annual incidence rates in human and annual numbers of livestock cases were plotted to show the overall temporal trend. The bar charts of average age- and gender-specific incidences were created during the study period, and the proportions of human cases by occupation were calculated. Demographic data from the 2010 census were used to calculate the average annual incidences for each county, standardized by the national distribution of age (10-year age-group categories) and sex (male and female).

The Kulldorff retrospective spatial-temporal and spatial-only scan statistics were used to detect human anthrax clustering areas at the county level (SaTScan software, version 9.3, http://www.satscan.org) [34]. The spatial-temporal statistic was calculated by forming the cylindrical windows with a geographic circle up to 50% of the population at risk and a time span up to 90% of the study period. The spatial-only scan statistic was calculated for each year using the maximum spatial cluster size of 10% of the population at risk. Clustering areas were identified using the log likelihood ratio (LLR) test based on a Poisson model, and the significance levels were evaluated using 999 Monte Carlo replications. A *P* value ≤ 0.05 was considered statistically significant.

A thematic map of the standardized average annual incidences of human anthrax was created, and the cumulative numbers of livestock anthrax cases and the identified spatial-temporal clustering areas were overlapped on the thematic map. To illustrate the spatiotemporal dynamics of human anthrax cases from 2005 to 2013, a bar chart of the annual numbers of human anthrax cases over the study period were shown for each province on the map. In addition, a map series for the spatial distribution of annual clustering areas and annual incidence rates of human anthrax were created. Spearman correlation was used to examine the association between monthly human anthrax incidence and each climatic variable (temperature, relative humidity, rainfall and sunshine hours) within the most likely clustering area using the Stata software, version 11.0 (StataCorp LP, Texas, USA) [35].

### Analysis of influencing factors of human anthrax occurrence

A boosted regression trees (BRT) model was applied to explore the potential determinants of spatial distribution of human anthrax at the county level. It is a method that combines the advantages of two algorithms, regression trees and boosting, and is able to accommodate non-linear relationships between outcomes and covariates and multiway interactions among covariates [36]. The weight of each variable estimated from all identified trees represents the influence of that variable in predicting the outcome. The BRT approach has been applied to the risk mapping for infectious diseases such as avian influenza A (H7N9), highly pathogenic avian influenza (H5N1) and dengue fever [37–40]. We used the data from 2005 to 2011 to construct the BRT model and the data from 2012 to 2013 to assess the model’s predictive power. We adopted a tree complexity of 5, a learning rate of 0.005 and a bag fraction of 75% to identify the optimal trees for each bootstrap data.

To address the issue of multicollinearity between climate variables, principal component analysis (PCA) was performed for these variables before the BRT modeling [41]. The principal component with the largest eigenvalue (>1.0) accounts for 84.61% of the total variability (S2 Table). We refer to this principal component as the meteorological index and included it as a covariate in the BRT model. Temperature, relative humidity, rainfall and sunshine hours contributed almost equally to the meteorological index, although only sunshine hours has a negative loading (S2 Table). The BRT modeling was carried out in sequential steps. First, 1,360 counties were randomly selected without replacement from all 2,650 counties without reported human anthrax cases throughout mainland China. These control counties were then combined with the 272 counties with human anthrax cases reported during 2005–2011 to form a bootstrap dataset with a 1-to-5 case-control ratio. Second, the bootstrap dataset was randomly portioned into a training dataset with 75% of the counties and a test dataset with 25%. Third, a BRT model was built using the training dataset and validated using the test dataset. Finally, the fitted model was used to predict the probability of human anthrax presence during 2012–2013 for all counties in mainland China. These steps were repeated for 50 times. Each time, a receiver-operating characteristic (ROC) curve was produced, and the area under the curve (AUC) was calculated to evaluate the predictive power of the model. A risk map of human anthrax infections in 2012–2013 was then created based on the average predicted probabilities of the 50 repetitions.

## Results

**Fig 1:**
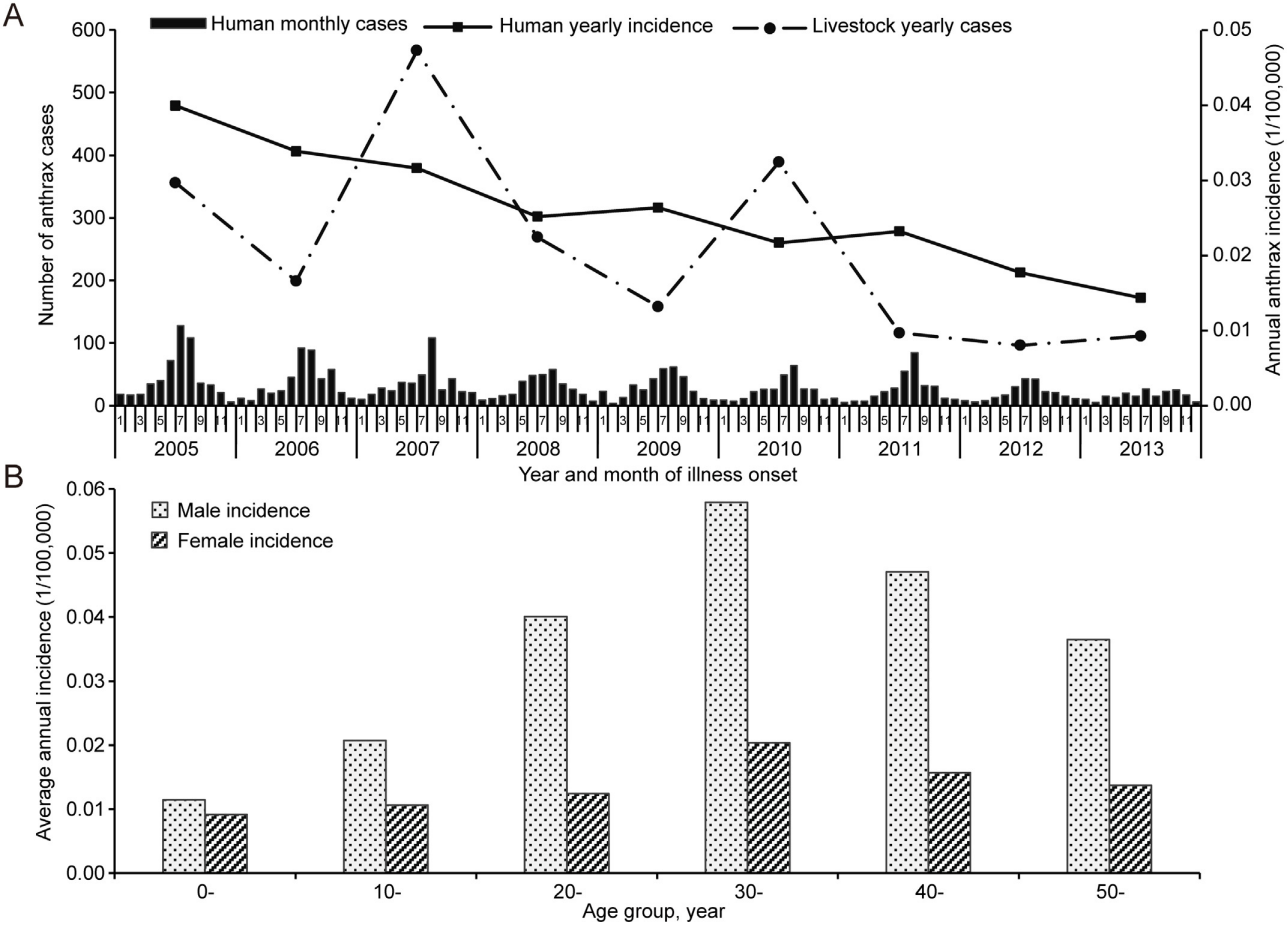
Temporal distribution and demographic profile of anthrax cases in mainland China during 2005–2013. (A) Temporal distribution of human and livestock cases of anthrax. (B) Average human anthrax incidences stratified by gender and age group.

A total of 3,115 human anthrax cases were reported in mainland China during 2005–2013. Cutaneous anthrax accounted for 97.7% of all the cases. The majority of the cases (72.2%) occurred in summer and autumn, and the disease usually peaked in July or August (Fig 1A). Males had a higher average annual incidence than females in the age groups of 20 years and above (Chi-square test, *P* < 0.01). The highest age-specific incidences were found in the age groups of 30–39 and 40–49 years for both males and females (Fig 1B). Herdsmen and peasants accounted for 88.7% of all cases reported during 2010–2013, followed by children less than 6 years old (31 cases, 3.0%) (Table 1). During 2005–2013, a total of 2,261 livestock anthrax cases were reported, the majority of which were cattle, sheep, goats and pigs (S3 Table). The epizootic curve of livestock anthrax was more fluctuating than the human epidemic curve but still showed an overall decreasing trend (Cochran-Armitage trend test, *P* < 0.01). It was moderately correlated with the epidemic of human anthrax, with a Spearman correlation coefficient of 0.38 (95% CI: 0.20–0.54, *P* < 0.01).

**Table 1:** Epidemiological characteristics of human anthrax cases in China, 2005–2013.

Characteristic	Total cases (n = 3115)
Demographic	
Male, No. (%)	2292 (73.58%)
The major age group (%)	30–49: 1423 (45.68%)
Peasant/herdsman, No. (%)^a^	909 (88.68%)
Temporal distribution	
2005	532
2006	451
2007	421
2008	335
2009	351
2010	289
2011	309
2012	236
2013	191
Spatial distribution	
No. of affected provinces	19
No. of affected counties	299
Most affected provinces (No.)	
Sichuan	917
Xinjiang	447
Guizhou	392
Gansu	370
Qinghai	225
Inner Mongolia	196

^**a**^The proportion of human cases according to occupation was calculated using data from 2010 to 2013.

**Fig 2:**
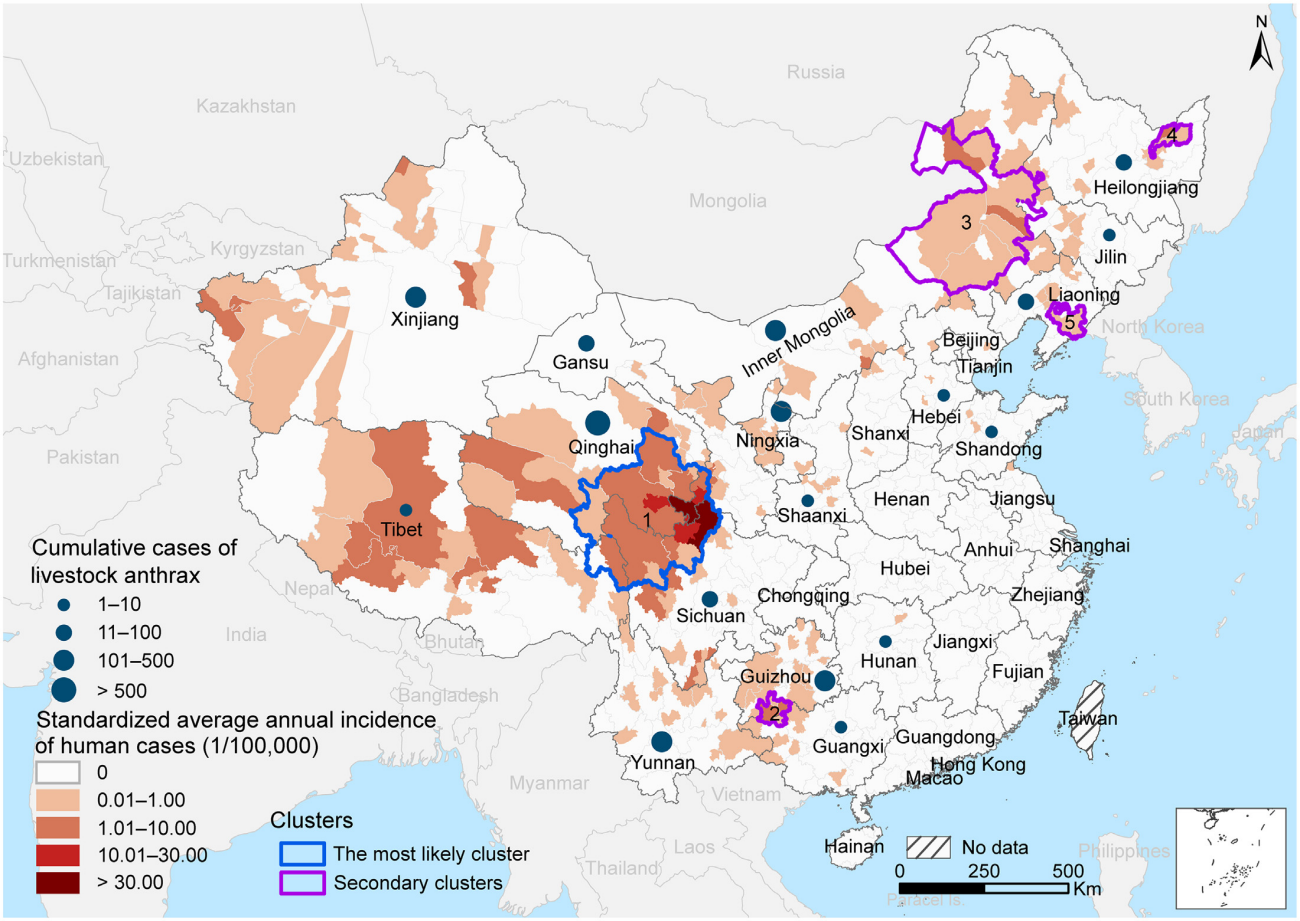
Standardized average annual incidences and spatial-temporal clusters of human anthrax at the county level. The cumulative numbers of livestock anthrax cases at the provincial level during 2005–2013 are overlapped.

Human anthrax cases were distributed in 299 counties of 19 provinces with an average annual incidence of 0.39 per 100, 000 person years (range: 0.01–51.98). About 82% of all cases were located in six provinces/autonomous regions of western and northeastern China, including Sichuan, Xinjiang, Guizhou, Gansu, Qinghai, and Inner Mongolia (Table 1). The remaining cases were distributed sporadically in 13 provinces/autonomous regions (Fig 2). Four provinces/autonomous regions, Gansu, Qinghai, Yunnan and Inner Mongolia, showed a rebound in the number of human cases in recent years, despite the overall decreasing trend in the whole China (S1 Fig). Qinghai is the province that suffered the most from livestock anthrax during the study period. The spatial distribution of human anthrax was mostly consistent with that of livestock anthrax, except for Sichuan Province (Fig 2, S1 Fig).

S2 Fig shows the dynamics of spatial clustering areas and standardized annual anthrax incidence of human anthrax. The most likely clusters were persistently located on the eastern Qinghai-Tibet Plateau, whereas the locations of secondary clusters varied over time. The spatiotemporal scan statistic identified one most likely cluster and four secondary clusters during the entire study period (2005–2013) (Fig 2). The most likely cluster consists of 34 counties on the junction of Sichuan, Gansu, Qinghai and Tibet provinces/autonomous regions, and spanned from January 2005 to January 2013, with a relative risk of 424.3 in comparison to counties outside the cluster. In the most likely cluster, monthly human anthrax incidence was positively correlated with monthly average temperature, relative humidity and monthly accumulative rainfall at lags of 0–2 months (S4 Table, S3 Fig). For the three climatic variables, the Spearman correlation coefficients ranged from 0.67 to 0.70 at lags of 0 or 1 month, and decreased with longer time lags. Sunshine hours at lags of 0–1 months were marginally correlated with monthly incidence of human anthrax (*P* = 0.048).

The BRT model found that the spatial distribution of human anthrax was significantly associated with the densities of cattle, sheep and human, coverage of meadow, coverage of typical grassland, elevation, coverage of topsoil with pH > 6.1, concentration of organic carbon in topsoil, and the meteorological index. All above potential predictors had weights (relative contribution) of more than 5.0 in the BRT models (Table 2). The maps of human anthrax incidence overlaid by these variables were also created to display the potential spatial association between them (S4 and S5 Figs). The probability of occurrence of human cases increased with higher values of densities of cattle and sheep, coverage of meadow, and concentration of organic carbon in topsoil. The risk rose quickly with higher elevations in the range of 500–1500 m, and plateaued or dropped for elevations above 1500m. In addition, the probability of occurrence of human anthrax cases was negatively associated with the density of human population, and the meteorological index (S6A Fig).

**Table 2:** Results of the boosted regression trees model applied to the human anthrax data in China during 2005–2011.

Variable	Boosted regression trees	
	Relative contribution (mean)	Relative contribution (sd)
Cattle density^b^	11.12	1.30
Sheep density^b^	8.18	1.21
Goats density	3.36	0.68
Human density^b^	8.04	1.88
Percentage coverage of meadow^b^	8.13	2.70
Percentage coverage of typical grassland^b^	5.55	1.21
Percentage coverage of alpine steppe	0.89	0.46
Elevation^b^	27.23	3.23
Percentage coverage of topsoil with pH > 6.1^b^	5.03	1.20
Average concentration of organic carbon^b^	9.87	1.54
Average concentration of calcium	3.30	0.86
Meteorological index^b^	9.30	1.75

^b^Variables reaching a weight (relative contribution) of more than 5 were considered significantly contributing to the occurrence of human anthrax infection.

**Fig 3:**
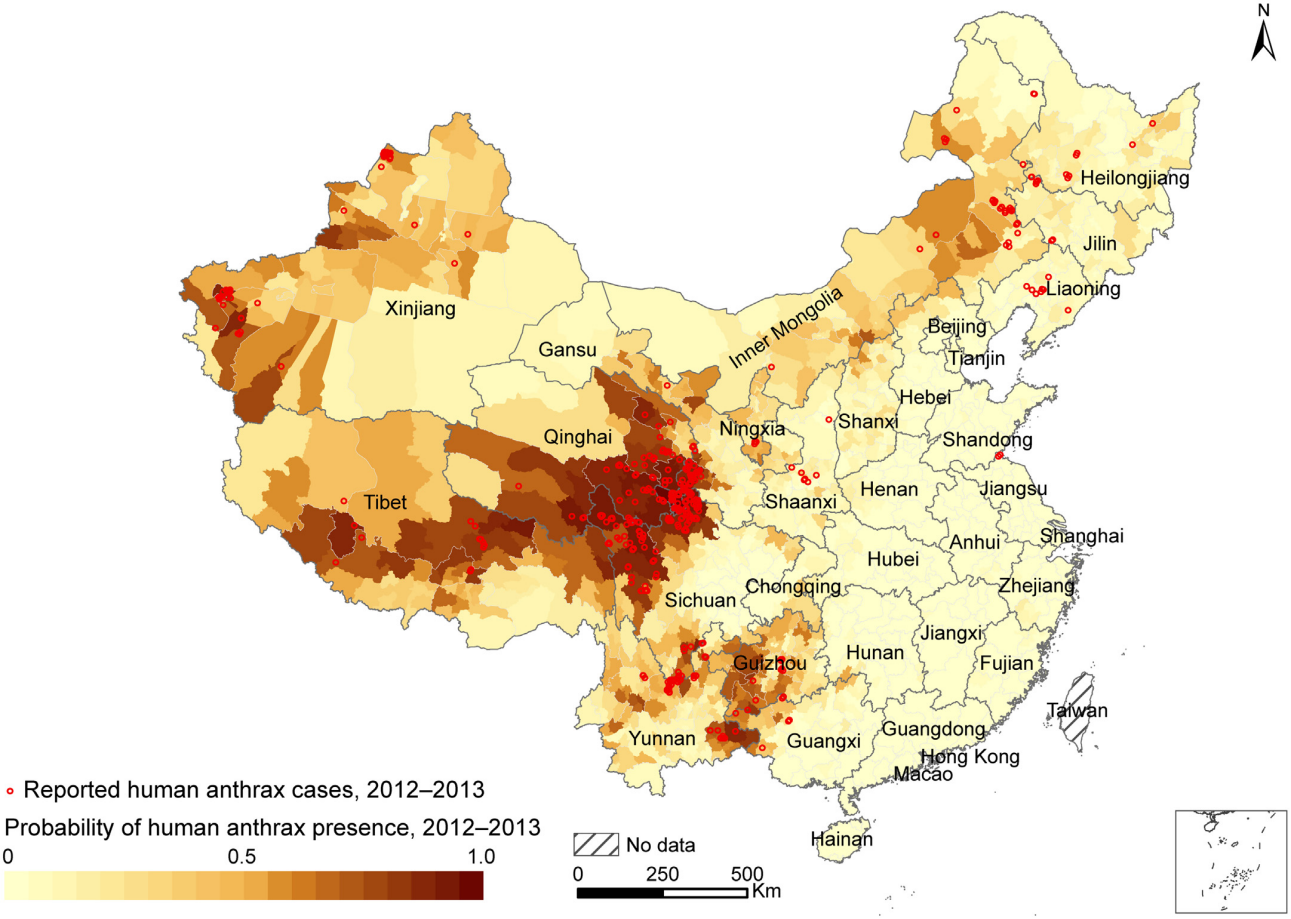
Predicted risks of human anthrax at the county level in China based on the BRT model and covariates for the period of 2012–2013. The BRT model was created and validated based on the data from 2005 to 2011, and the red circles represent the reported human anthrax cases from 2012 to 2013.

The estimated AUC value of 0.921 (95% CI: 0.899–0.943) indicates a decent predictive power for the probability of occurrence of human cases. The AUC estimates are 0.975 (95% CI: 0.966–0.984) and 0.892 (95% CI: 0.873–0.912) for the training and test dataset, respectively (S6B Fig). To avoid overfitting of the model, which is possible considering the enormous heterogeneity in all variables across the whole country, we performed a sensitivity analysis by restricting case counties and the sampling of control counties to seven provinces (Sichuan, Qinghai, Gansu, Guizhou, Inner Mongolia, Heilongjiang and Liaoning provinces /autonomous regions) where spatiotemporal clusters of the disease were located. The resulted ROC and AUC’s are similar to our final model based on the data of the whole country (S6C Fig). On the basis of the average predicted probability of occurrence of human cases for each county in 2012–2013, the high-risk areas of human anthrax were mainly distributed in four regions: (1) the central-west high-risk region that contains most of the Qinghai-Tibetan Plateau, and covers eastern Qinghai, northwestern Sichuan, southwestern Gansu, and central Tibet; (2) the southwest high-risk region that consists of Yunnan, Guizhou and western Guangxi provinces; (3) the northwest high-risk region that covers western and northwestern Xinjiang; and (4) the north high-risk region that covers central and eastern Inner Mongolia, western and eastern Heilongjiang, and Jilin provinces (Fig 3). By superimposing the locations of reported human anthrax cases on the predictive risk map during 2012–2013, we found that 93.1% of reported cases were located in the high risk counties each with a probability of occurrence of human cases more than 0.7. Coinciding with the most likely cluster, the eastern part of the central-west high-risk region has the highest risk of occurrence of human cases.

## Discussion

Our study provides a complete overview of spatiotemporal dynamics of human and livestock anthrax in mainland China from 2005 to 2013. It is the first national risk assessment of human anthrax occurrence in China. Our study identified five clustering areas of human anthrax cases and four potential high-risk regions for the occurrence of human cases. In addition, we quantified the relationship of climate factors to the temporal trend of human anthrax and the contribution of agro-ecological, environmental and meteorological factors to the spatial distribution of human anthrax.

We found that males had a higher incidence than females, in particular for adults, probably due to the occupational exposure. Among herders and farmers, men are usually more exposed to livestock than women by undertaking most agricultural activities such as pasturing and slaughtering [13,14]. Although incidences of human cases and livestock cases were significantly correlated, inconsistency was found in their spatial distributions in some provinces, e.g., Tibet and Sichuan, likely due to underreporting of anthrax case in livestock or the possibility that more than one person may contract the disease from a single animal [42]. A serological surveillance study carried out by the Chinese Institute of Epidemiology and Microbiology during 1990–1994 showed that 29.1% of human samples and 31.2% of livestock samples had detectable antibodies to the capsular of *Bacillus anthracis* at outbreak spots [43]. Since then, there have been very few studies on serological prevalence of anthrax both in human and livestock across the country. It is necessary to strengthen the surveillance of anthrax in livestock, especially in the four high-risk regions identified in this study.

Densities of cattle and sheep were identified as useful predictors for the risk of human anthrax. Suitable habitat conditions for these livestock were also important predictors, i.e., a higher coverage of meadow, a higher elevation or a lower human density. Unlike brucellosis (another zoonotic disease) [44], the presence of human anthrax was not found to be associated with the density of goats. This could be partially explained by the difference in the feeding habits between goats and other livestock. It was documented that cattle ingest lots of soil by pulling the plant out of the ground when grazing, whereas goats usually browse on grass only, which makes them less exposed to spores in soil [5, 21]. There were only two outbreaks of anthrax among goats during the study period, as compared to 107 outbreaks in cattle and 21 in sheep (S3 Table).

The ability of *Bacillus anthracis* to form long-lasting, highly resistant spores is the key to the persistence of anthrax in any area [22]. Certain soil characteristics, such as high levels of organic matter, pH or calcium, were thought to facilitate the survival of spores [5, 21, 22, 45], although the role of the calcium level was not always clear [46]. We found the evidence for the ecological association of human anthrax with the pH value of topsoil and the concentration of organic carbon in topsoil (Table 2, S5C and S5D Fig), but the concentration of calcium in topsoil was not picked by our model (Table 2, S5E Fig). In our data, the presence of human anthrax was largely driven by the distribution of livestock and suitable habitats for them, and the calcium level in the topsoil of the habitats is generally lower than that of non-epidemic areas, as shown in S5E Fig. However, the possibility of spatial heterogeneity in the effect of the calcium level on the presence of the disease cannot be ruled out.

Temperature, relative humidity and rainfall were positively correlated over time with human anthrax in the most likely clustering area (S4 Table, S3 Fig). Increased rainfall and temperature in the summer could unearth the anthrax spores and facilitate the breeding of vectors, such as tabanids and *Stomoxys* [5, 21, 47, 48]. Moreover, the transfer of *Bacillus anthracis* between the vegetative form and the spore was thought to be related to temperature and relative humidity [49]. However, the exact ecological mechanism of climatic influence on the seasonality of anthrax is not clear and may vary across geographic regions. For example, drought easily makes the herbivores more exposed to the spores in soil by inhibiting the growth of grass [5, 21]. Extreme temperatures may also depress innate immunity of the host, reducing the minimal dose of anthrax for infection [5, 47]. In contrast to the positive temporal association of the climate variables to the incidence of the disease, it is interesting that the spatial risk distribution of human anthrax was negatively associated with the meteorological index and thus negatively associated with average temperature, relative humidity and rainfall. The negative association in the spatial dimension and the positive association in the temporal dimension at any given location do not contradict each other. In addition, the spatial association was partially due to the relatively high elevations of livestock habitats where average annual temperature, relative humidity and rainfall are relatively low.

Our results should be interpreted with the following limitations in mind. First, human and livestock cases could have been under-reported as the surveillance was passive. The changes in the diagnostic criteria for human anthrax cases since 2008 might have affected the quantity of the reported data. Second, the BRT model is an ecological analysis of relative contributions of risk factors and offers no causal interpretation. The causality of the identified risk factors can be tested with appropriately designed studies in the future. Third, some relevant risk factors were not available to refine our exploration, including but not limited to seroprevalence in human and livestock, exposure level of people at risk, and the industrialization level of livestock production [21].

Although anthrax underwent an overall decreasing trend during the study period, incidences rebounded and outbreaks were reported in recent years in some provinces [27–31]. While imposing threats to both animal productivity and human health in affected communities, anthrax remains a largely neglected zoonosis [50]. Existing surveillance programs for anthrax should be improved and expanded to cover livestock, human, and environmental samples, a “One Health” approach [14, 51, 52]. In addition, vaccination of both livestock and human would be essential for disease prevention and can be prioritized for high-risk regions identified in our work [42, 53].

## Supporting Information

S1 Text The case definitions of human and livestock anthrax. (DOCX)

S1 Table Description of risk factors used in the spatial analyses. (DOCX)

S2 Table Details about the principal component analysis on climatic variables. (DOCX)

S3 Table Outbreaks of the livestock anthrax in mainland China, 2005–2013. (DOCX)

S4 Table Spearman correlation coefficients (95% confidence interval) between monthly incidence of human anthrax and climate variables within the most likely cluster. (DOCX)

S1 Fig The distribution of human and livestock anthrax cases at province level in mainland China, 2005–2013. (TIF)

S2 Fig The maps of human anthrax cases from 2005 to 2013 overlaid with yearly spatial clusters in mainland China, 2005–2013. (TIF)

S3 Fig The temporal relationship between monthly anthrax incidence and climate variables within the most likely cluster. (TIF)

S4 Fig The maps of human anthrax cases from 2005 to 2013 overlaid with the distributions of anthropogenic factors in mainland China. (A) Cattle density; (B) Sheep density; (C) Goats density; (D) Human density.
(TIF)

S5 Fig The maps of human anthrax cases from 2005 to 2013 overlaid with environmental factors in mainland China. (A) Land cover; (B) Elevation; (C) pH in topsoil; (D) Concentration of organic carbon in topsoil; (E) Concentration of calcium in topsoil; (F) Monthly average temperature during the study period; (G) Monthly average relative humidity during the study period; (H) Yearly accumulative rainfall during the study period; (I) Yearly accumulative sunshine hours during the study period.
(TIF)

S6 Fig The outcomes based on the BRT models. (A) Relationships between the risk of human anthrax occurrence and risk factors (contribution weights ≥ 5) based on the BRT model; (B) ROC curves for the BRT models built on national data; (C) ROC curves for the BRT models built on counties in the seven provinces that reported human anthrax cases. ROC curves for all 50 bootstrap datasets are colored in grey. The average ROCs based on the training set, the test set and the prediction set (2012–2013) are colored in blue, red and black, respectively.
(TIF)
