# Correction: Mapping the Distribution of Anthrax in Mainland China, 2005–2013

**DOI:** 10.1371/journal.pntd.0004708

**Published:** 2016-05-04

**Authors:** The PLOS Neglected Tropical Diseases Staff

There are errors in the Funding section. The correct funding information is as follows: The study was supported by the Special Program for Prevention and Control of Infectious Diseases in China (No. 2013ZX10004218), the Basic Work on Special Program for Science & Technology Research (2013FY114600), Y Yang was supported by the US National Institutes of Health grant for Modeling of Infectious Disease Agent Study Center for Excellence (grant U54-GM111274), HJ Yu is funded by the Ministry of Science and Technology, China (grants 2012ZX10004-201 and 2014BAI13B05), the China CDC’s Key Laboratory of Surveillance and Early-warning on Infectious Disease, grants from the US National Institutes of Health (Comprehensive International Program for Research on AIDS grant U19 AI51915) and the National Science Fund for Distinguished Young Scholars (No. 81525023), and SJ Lai is funded by the Flowminder Foundation for his postgraduate research on population movement and infectious disease dynamics in the University of Southampton. The funders had no role in study design,data collection and analysis, decision to publish, or preparation of the manuscript.

## References

[1] Chen W-J, Lai S-J, Yang Y, Liu K, Li X-L, Yao H-W, et al (2016) Mapping the Distribution of Anthrax in Mainland China, 2005–2013. PLoS Negl Trop Dis 10(4): e0004637 doi:10.1371/journal.pntd.0004637 27097318 10.1371/journal.pntd.0004637 PMC4838246

