# Supplementary material for: Mapping the Distribution of Anthrax in Mainland China, 2005–2013
Source: PLoS Negl Trop Dis. 2016 Apr 20;10(4):e0004637. doi: 10.1371/journal.pntd.0004637 (PMC4838246; doi:10.1371/journal.pntd.0004637)
Supplement: S1 Text — (DOCX) [file pntd.0004637.s001.docx]

**S1 Text. The case definitions of human and livestock anthrax**

The case definition of human anthrax was published by Chinese Ministry of Health. A clinically-diagnosed case was defined as a case with an anthrax-linked epidemiological history, presentation of clinical manifestations, and the demonstration of *Bacillus anthracis* in a clinical specimen by microscopic examination of stained smears. A laboratory-confirmed case was defined as a clinically-diagnosed case plus laboratory evidence of *Bacillus anthracis* infection detected by bacteria isolation, or a 4-fold increase in titer of specific antibody in blood sera collected from the acute and convalescent stages of illness. Epidemiological history includes living in areas with confirmed anthrax cases or travelling to such areas within 14 days prior to the onset of illness or engagement in occupations that are likely to result in exposure to anthrax. Clinical manifestations include cutaneous, gastrointestinal, or inhalational anthrax plus meningitis and septicemia. Physicians in medical institutions made diagnosis according to the national standards of “Diagnostic Criteria and Principles of Management for Anthrax (GB 17015–1997)” before the year of 2008 and “Diagnostic Criteria for Anthrax (WS 283–2008)” thereafter.

The case definition of livestock anthrax was published by The Ministry of Agriculture of the People's Republic of China. Diagnoses were generally made based on the combination of epidemiological investigations, clinical manifestations of different animals, changes in pathology, and laboratory evidence of *Bacillus anthracis* infection. Relevant information can be found in the government-issued documents titled “Diagnostic Techniques for Animal Anthrax (NY/T 561–2002)” and “Guideline of Prevention and Treatment Techniques for Animal Anthrax”.
