# Supplementary material for: Mapping the Distribution of Anthrax in Mainland China, 2005–2013
Source: PLoS Negl Trop Dis. 2016 Apr 20;10(4):e0004637. doi: 10.1371/journal.pntd.0004637 (PMC4838246; doi:10.1371/journal.pntd.0004637)
Supplement: S1 Table — (DOCX) [file pntd.0004637.s002.docx]

**S1 Table. Description of risk factors used in the spatial analyses.**

| Variables | Description | Transformation | Type |
| --- | --- | --- | --- |
| Cattle density | Density of cattle (heads per km^2^) | Log_10_(x+1) | Continuous |
| Sheep density | Density of sheep (heads per km^2^) | Log_10_(x+1) | Continuous |
| Goats density | Density of goats (heads per km^2^) | Log_10_(x+1) | Continuous |
| Human density | Density of population (persons per km^2^) | Log_10_(x+1) | Continuous |
| Coverage of meadow | Percentage coverage of meadow (1%) | - | Continuous |
| Coverage of typical grassland | Percentage coverage of typical grassland (1%) | - | Continuous |
| Coverage of alpine steppe | Percentage coverage of alpine steppe (1%) | - | Continuous |
| Elevation | Average elevation (m) | - | Continuous |
| Coverage of topsoil with pH > 6.1 | Percentage coverage of topsoil with pH > 6.1 (1%) | - | Continuous |
| Concentration of organic carbon | Average concentration of organic carbon in topsoil (1%) | - | Continuous |
| Concentration of calcium | Average concentration of calcium compounds in topsoil (1% CaCO_3_) | - | Continuous |
| Temperature | Monthly average temperature during the study period (℃) | - | Continuous |
| Relative humidity | Monthly average relative humidity during the study period (1%) | - | Continuous |
| Rainfall | Yearly accumulative rainfall during the study period (mm) | - | Continuous |
| Sunshine hours | Yearly accumulative sunshine hours during the study period (1%) | - | Continuous |
