# Supplementary material for: Mapping the Distribution of Anthrax in Mainland China, 2005–2013
Source: PLoS Negl Trop Dis. 2016 Apr 20;10(4):e0004637. doi: 10.1371/journal.pntd.0004637 (PMC4838246; doi:10.1371/journal.pntd.0004637)
Supplement: S2 Table — (DOCX) [file pntd.0004637.s003.docx]

**S2 Table. Details about the principal component analysis on climatic variables.** Values outside and inside the parentheses represent the results for the periods of 2005–2011 and 2012–2013, respectively.

|  | | Component 1 | Component 2 | Component 3 | Component 4 |
| --- | --- | --- | --- | --- | --- |
| Eigenvalue | | 3.38 (3.29) | 0.33 (0.34) | 0.21 (0.27) | 0.07 (0.10) |
| Cumulative contribution (%) | | 84.61 (82.36) | 92.94 (90.77) | 98.15 (97.48) | 100.00 (100.00) |
| Loadings | Temperature | 0.49 (0.48) | 0.64 (0.68) | -0.49 (-0.52) | 0.35 (0.20) |
|  | Relative humidity | 0.51 (0.52) | -0.41 (-0.38) | 0.42 (0.26) | 0.63 (0.72) |
|  | Rainfall | 0.51 (0.50) | 0.34 (0.29) | 0.56 (0.68) | -0.56 (-0.45) |
|  | Sunshine hours | -0.49 (-0.50) | 0.55 (0.56) | 0.53 (0.45) | 0.41 (0.48) |
