# Supplementary material for: Mapping the Distribution of Anthrax in Mainland China, 2005–2013
Source: PLoS Negl Trop Dis. 2016 Apr 20;10(4):e0004637. doi: 10.1371/journal.pntd.0004637 (PMC4838246; doi:10.1371/journal.pntd.0004637)
Supplement: S3 Table — (DOCX) [file pntd.0004637.s004.docx]

**S3 Table. Outbreaks of the livestock anthrax in mainland China, 2005–2013.**

| Livestock | No. of cases | No. of outbreaks |
| --- | --- | --- |
| Cattle | 927 | 107 |
| Sheep | 316 | 21 |
| Goats | 425 | 2 |
| Pigs | 262 | 34 |
| Horses | 1 | 1 |
| Cattle & sheep ^ơ^ | 209 | 32 |
| Cattle & pigs ^ơ^ | 13 | 1 |
| Pigs & sheep ^ơ^ | 27 | 2 |
| Cattle & horses ^ơ^ | 68 | 7 |
| Others | 13 | 2 |

^ơ^Outbreaks whose information about species were mixed together and

couldn’t be figured out.
