# Supplementary material for: Mapping the Distribution of Anthrax in Mainland China, 2005–2013
Source: PLoS Negl Trop Dis. 2016 Apr 20;10(4):e0004637. doi: 10.1371/journal.pntd.0004637 (PMC4838246; doi:10.1371/journal.pntd.0004637)
Supplement: S4 Table — (DOCX) [file pntd.0004637.s005.docx]

**S4 Table. Spearman correlation coefficients (95% confidence interval) between monthly incidence of human anthrax and climate variables within the most likely cluster.**

| Time lags (month) | Temperature | Relative humidity | Rainfall | Sunshine hours |
| --- | --- | --- | --- | --- |
| 0 | 0.67^*^ | 0.67^*#^ | 0.67^*^ | -0.19^*^ |
|  | 0.53 to 0.78 | 0.54 to 0.77 | 0.54 to 0.78 | -0.39 to 0.01 |
| 1 | 0.70^*#^ | 0.54^*^ | 0.68^*#^ | -0.19^*^ |
|  | 0.58 to 0.79 | 0.40 to 0.66 | 0.55 to 0.78 | -0.38 to 0.01 |
| 2 | 0.50^*^ | 0.28^*^ | 0.50^*^ | -0.03 |
|  | 0.37 to 0.61 | 0.11 to 0.42 | 0.36 to 0.63 | -0.21 to 0.17 |
| 3 | 0.18 | -0.08 | 0.17 | 0.22^*#^ |
|  | 0.01 to 0.33 | -0.26 to 0.11 | -0.01 to 0.33 | 0.04 to 0.40 |

^*^Variables with *P* value ≤ 0.05 were considered significantly correlated with the monthly human anthrax incidence.

^#^Maximum value of Spearman correlation coefficients for each variable.
