# Supplementary figures and images for: Mapping the Distribution of Anthrax in Mainland China, 2005–2013
Source: PLoS Negl Trop Dis. 2016 Apr 20;10(4):e0004637. doi: 10.1371/journal.pntd.0004637 (PMC4838246; doi:10.1371/journal.pntd.0004637)

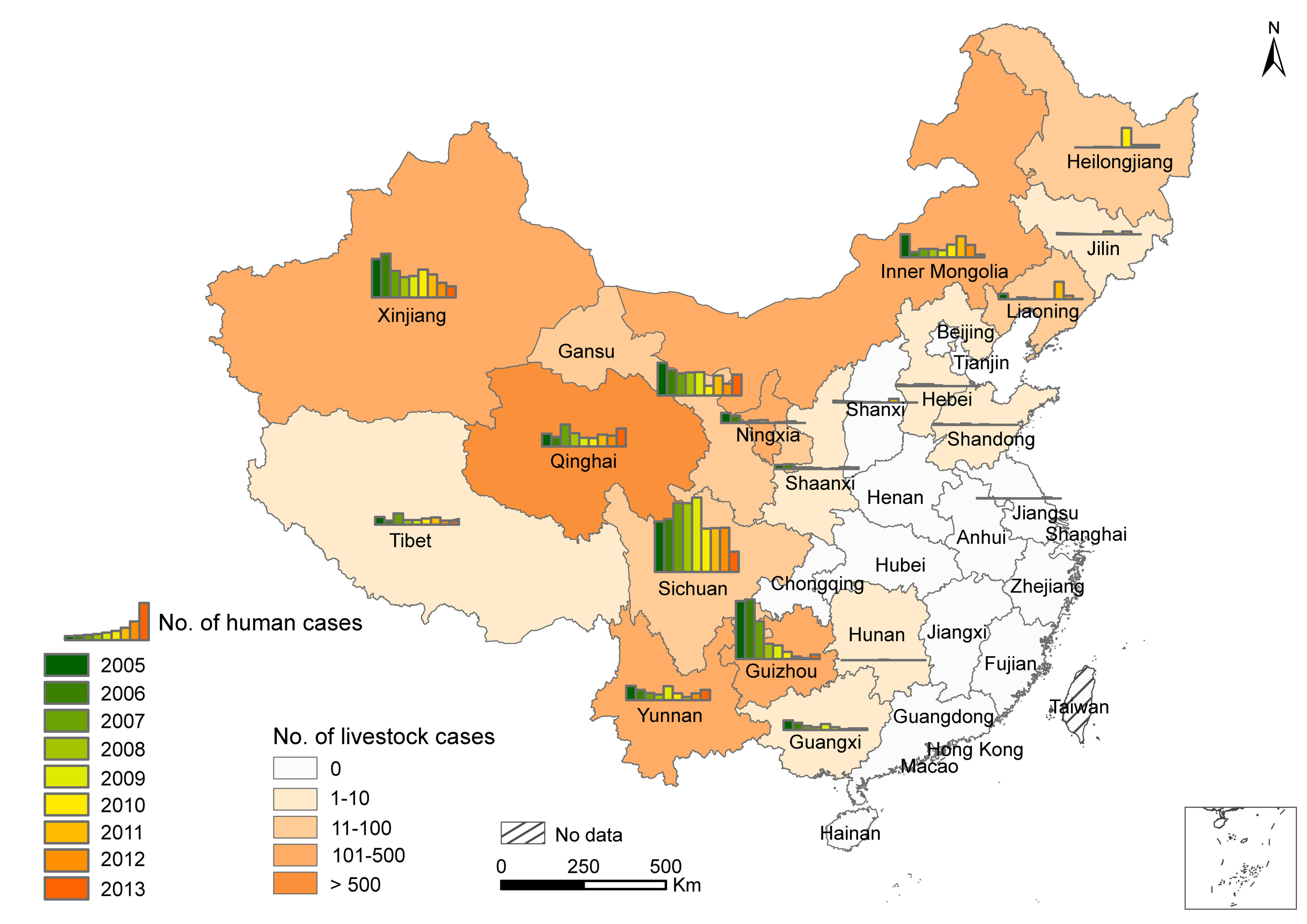

Supplement: S1 Fig — (TIF) [file pntd.0004637.s006.tif]

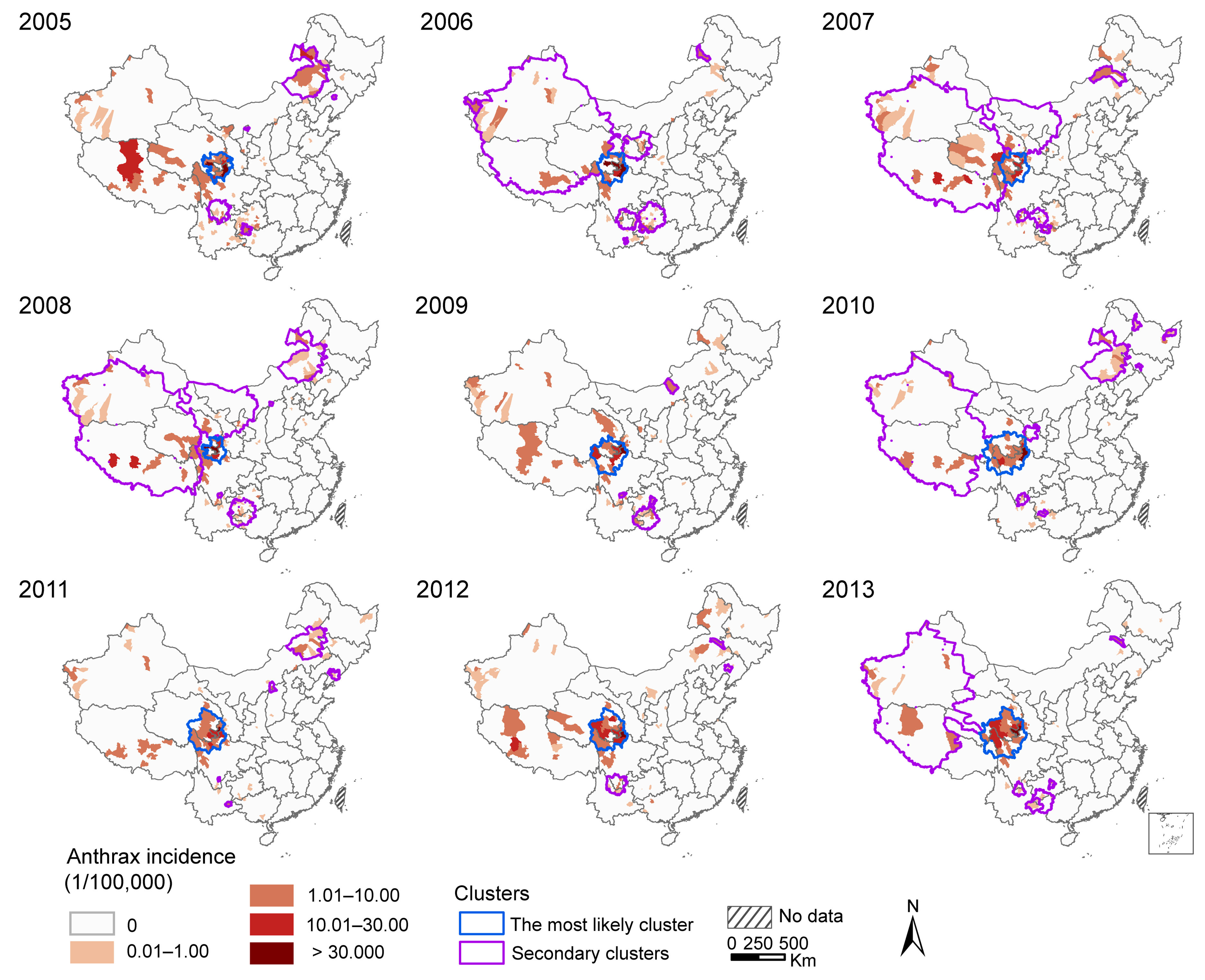

Supplement: S2 Fig — (TIF) [file pntd.0004637.s007.tif]

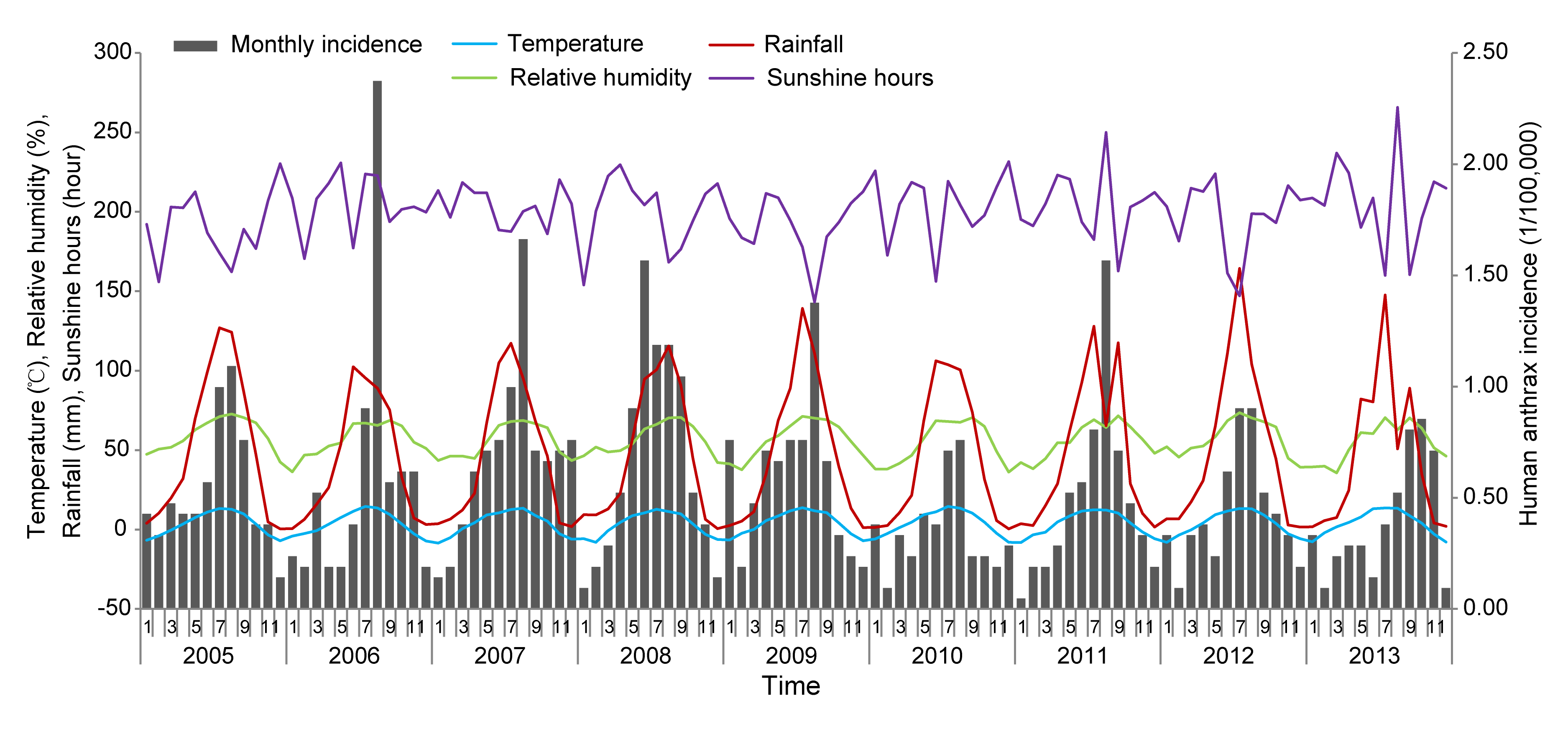

Supplement: S3 Fig — (TIF) [file pntd.0004637.s008.tif]

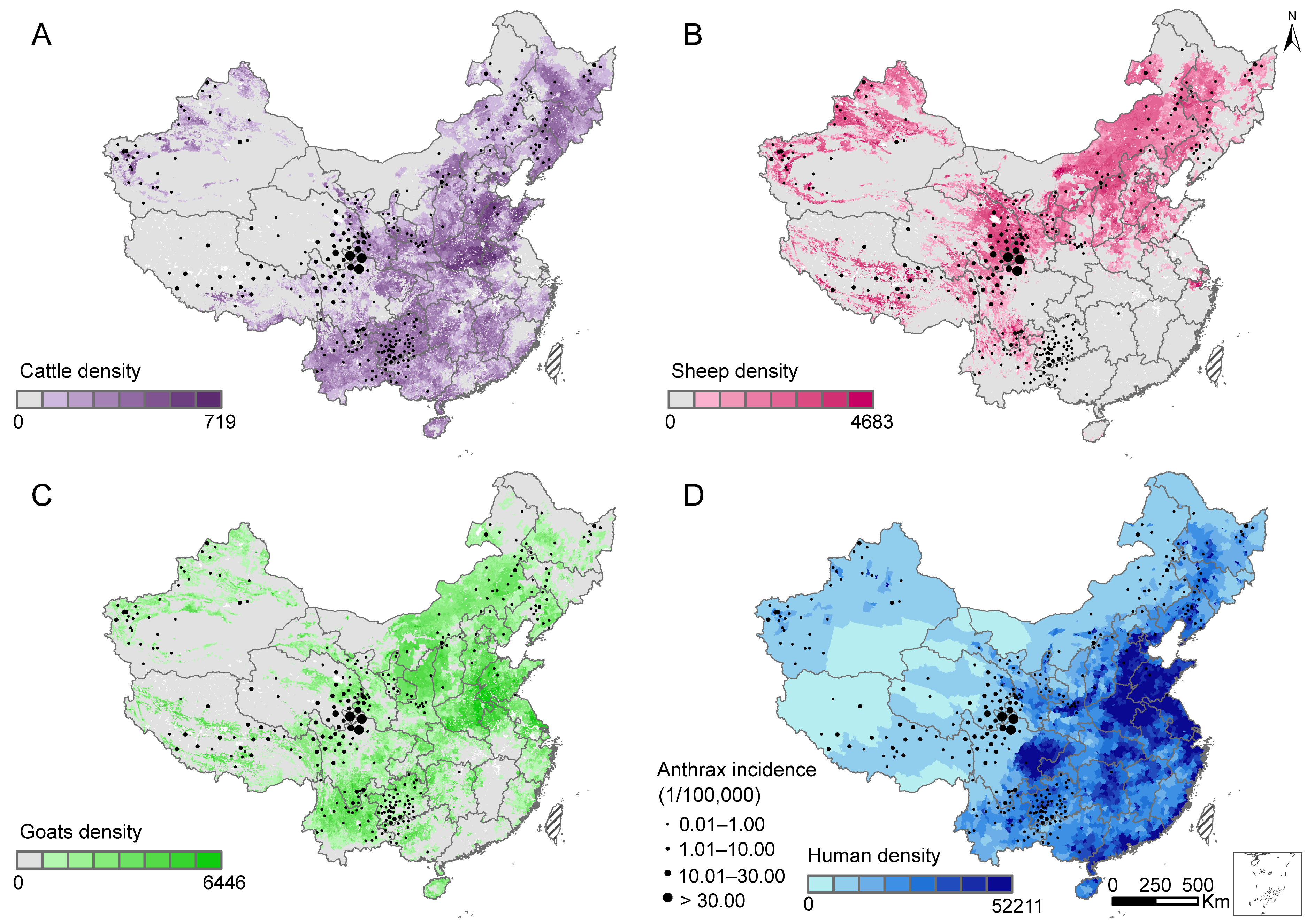

Supplement: S4 Fig — (A) Cattle density; (B) Sheep density; (C) Goats density; (D) Human density. (TIF) [file pntd.0004637.s009.tif]

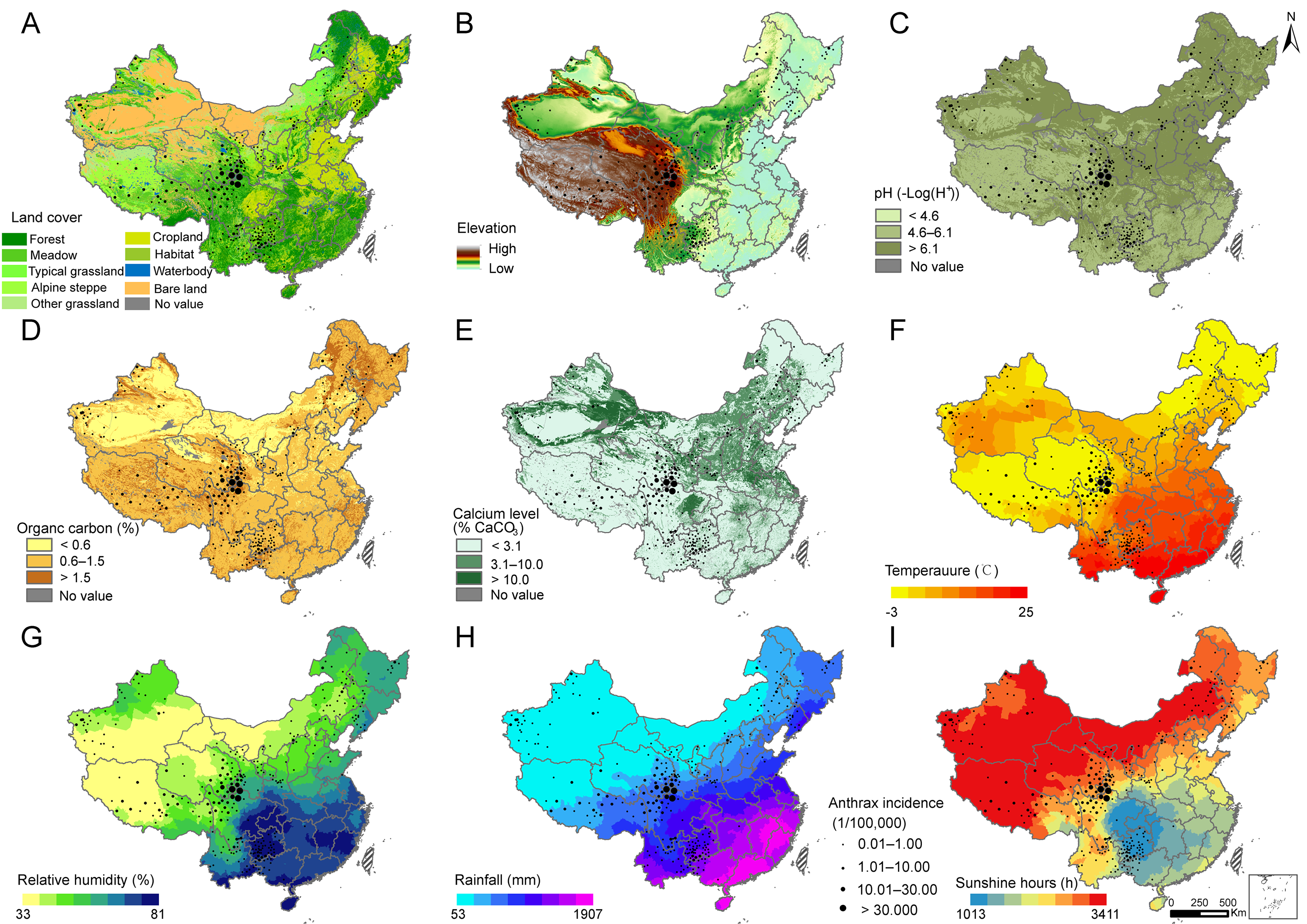

Supplement: S5 Fig — (A) Land cover; (B) Elevation; (C) pH in topsoil; (D) Concentration of organic carbon in topsoil; (E) Concentration of calcium in topsoil; (F) Monthly average temperature during the study period; (G) Monthly average relative humidity during the study period; (H) Yearly accumulative rainfall during the study period; (I) Yearly accumulative sunshine hours during the study period. (TIF) [file pntd.0004637.s010.tif]

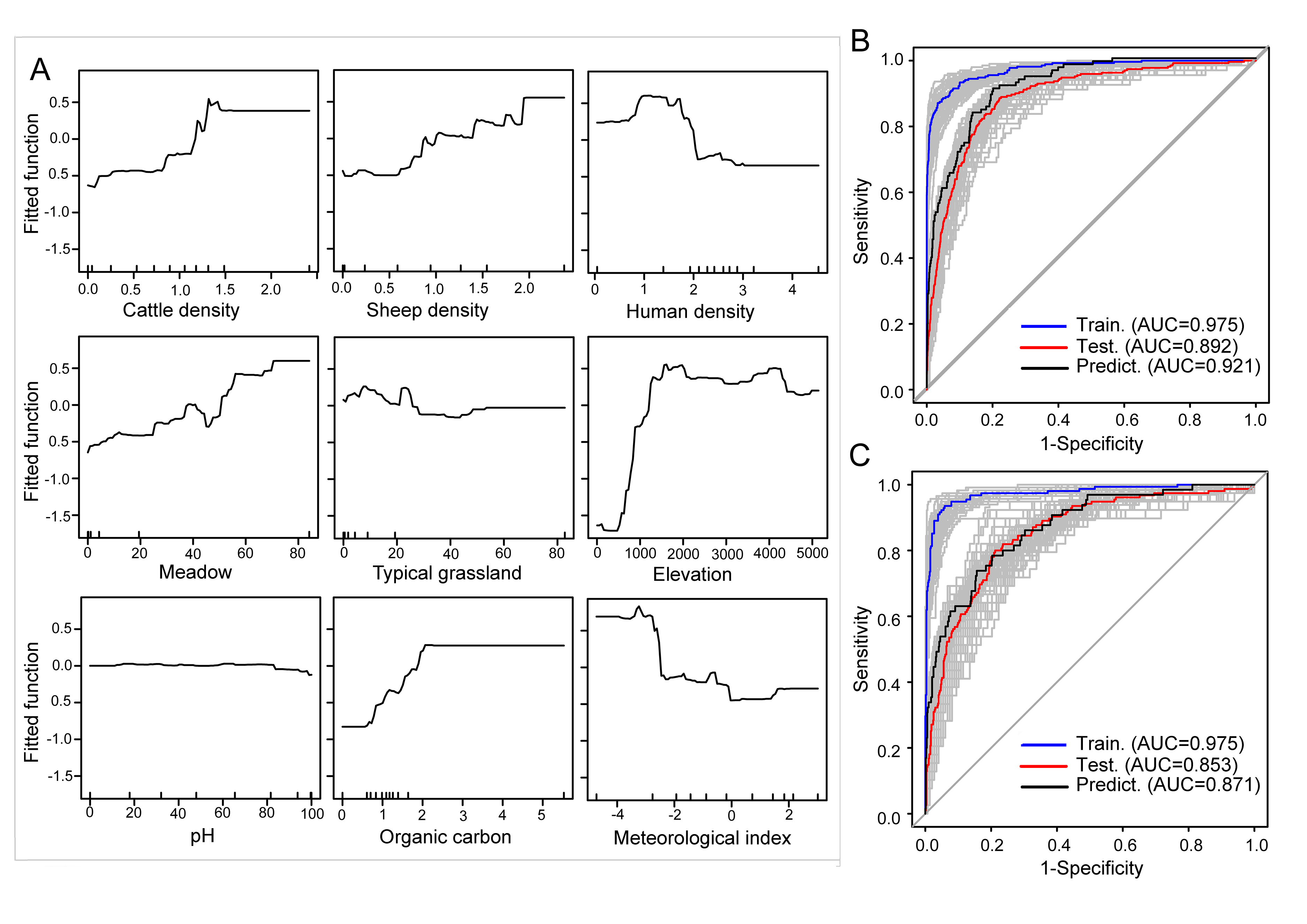

Supplement: S6 Fig — (A) Relationships between the risk of human anthrax occurrence and risk factors (contribution weights ≥ 5) based on the BRT model; (B) ROC curves for the BRT models built on national data; (C) ROC curves for the BRT models built on counties in the seven provinces that reported human anthrax cases. ROC curves for all 50 bootstrap datasets are colored in grey. The average ROCs based on the training set, the test set and the prediction set (2012–2013) are colored in blue, red and black, respectively. (TIF) [file pntd.0004637.s011.tif]
